# Aberrant patterns of PET response during treatment for DLBCL patients with *MYC* gene rearrangements

**DOI:** 10.1007/s00259-021-05498-7

**Published:** 2021-09-02

**Authors:** J. J. Eertink, A. I. J. Arens, J. E. Huijbregts, F. Celik, B. de Keizer, S. Stroobants, D. de Jong, S. E. Wiegers, G. J. C. Zwezerijnen, C. N. Burggraaff, R. Boellaard, H. C. W. de Vet, O. S. Hoekstra, P. J. Lugtenburg, M. E. D. Chamuleau, J. M. Zijlstra, the HOVON imaging workgroup

**Affiliations:** 1 Department of Hematology, Cancer Center Amsterdam, Amsterdam UMC, Vrije Universiteit Amsterdam, De Boelelaan 1117, 1081 HV Amsterdam, The Netherlands; 2 Department of Radiology, Nuclear Medicine and Anatomy, Radboud University Medical Center, Geert Grooteplein Zuid 10, Nijmegen, The Netherlands; 3 Department of Radiology and Nuclear Medicine, Gelre Ziekenhuizen, Albert Schweitzerlaan 31, Apeldoorn, The Netherlands; 4 Department of Radiology and Nuclear Medicine, Deventer Ziekenhuis, Nico Bolkesteinlaan 75, Deventer, The Netherlands; 5 Department of Radiology and Nuclear Medicine, University Medical Center Utrecht, Heidelberglaan 100, Utrecht, The Netherlands; 6 Department of Nuclear Medicine, Antwerp University Hospital (UZA), Antwerp, Belgium; 7 Department of Pathology, Cancer Center Amsterdam, Amsterdam UMC, Vrije Universiteit Amsterdam, de Boelelaan 1117, Amsterdam, The Netherlands; 8 Department of Radiology and Nuclear Medicine, Cancer Center Amsterdam, Amsterdam UMC, Vrije Universiteit Amsterdam, de Boelelaan 1117, Amsterdam, The Netherlands; 9 Department of Epidemiology and Data Science, Amsterdam Public Health Research Institute, Amsterdam UMC, Vrije Universiteit Amsterdam, de Boelelaan 1117, Amsterdam, The Netherlands; 10 Department of Hematology, Erasmus MC Cancer Institute, University Medical Center Rotterdam, Wytemaweg 80, Rotterdam, The Netherlands

**Keywords:** 18F FDG PET/CT, Diffuse large B-cell lymphoma, MYC rearrangement, Response, Deauville score

## Abstract

**Purpose:**

*MYC* gene rearrangements in diffuse large B-cell lymphoma (DLBCL) patients are associated with poor prognosis. Our aim was to compare patterns of 2[^18^F]fluoro-2-deoxy-D-glucose positron emission tomography computed tomography (PET/CT) response in *MYC* + and *MYC*- DLBCL patients.

**Methods:**

Interim PET/CT (I-PET) and end of treatment PET/CT (EoT-PET) scans of 81 *MYC* + and 129 *MYC*- DLBCL patients from 2 HOVON trials were reviewed using the Deauville 5-point scale (DS). DS1-3 was regarded as negative and DS4-5 as positive. Standardized uptake values (SUV) and metabolic tumor volume (MTV) were quantified at baseline, I-PET, and EoT-PET. Negative (NPV) and positive predictive values (PPV) were calculated using 2-year overall survival.

**Results:**

*MYC* + DLBCL patients had significantly more positive EoT-PET scans than *MYC*- patients (32.5 vs 15.7%, *p* = 0.004). I-PET positivity rates were comparable (28.8 vs 23.8%). In *MYC* + patients 23.2% of the I-PET negative patients converted to positive at EoT-PET, vs only 2% for the *MYC*- patients (*p* = 0.002). Nine (34.6%) *MYC* + DLBCL showed initially uninvolved localizations at EoT-PET, compared to one (5.3%) *MYC*- patient. A total of 80.8% of EoT-PET positive *MYC* + patients showed both increased lesional SUV and MTV compared to I-PET. In *MYC-* patients, 31.6% showed increased SUV and 42.1% showed increased MTV. NPV of I-PET and EoT-PET was high for both *MYC* subgroups (81.8–94.1%). PPV was highest at EoT-PET for *MYC* + patients (61.5%).

**Conclusion:**

*MYC* + DLBCL patients demonstrate aberrant PET response patterns compared to MYC- patients with more frequent progression during treatment after I-PET negative assessment and new lesions at sites that were not initially involved.

**Trial registration number and date of registration:**

HOVON-84: EudraCT: 2006–005,174-42, retrospectively registered 01–08-2008. HOVON-130: EudraCT: 2014–002,654-39, registered 26–01-2015

**Supplementary Information:**

The online version contains supplementary material available at 10.1007/s00259-021-05498-7.

## Introduction

Diffuse large B-cell lymphoma (DLBCL) is the most common subtype of non-Hodgkin lymphoma with heterogeneous outcome. This heterogeneity can partially be explained by genetic abnormalities, such as *MYC* oncogene rearrangements, that occur in approximately 10–15% of DLBCL patients and predict outcome independently [1–3]. *MYC* plays a central role in many aspects of the oncogenic process, coordinating metabolism, proliferation, apoptosis, and differentiation of cells. *MYC* rearrangement is associated with poor outcomes upon standard first-line rituximab, cyclophosphamide, doxorubicin, vincristine, and prednisone (R-CHOP) therapy [1–3]. Approximately 30% of *MYC* + large B-cell lymphoma patients have a *MYC* rearrangement (single hit (SH) DLBCL), whereas in 70% *MYC* rearrangements are detected together with *BCL2* and/or *BCL6* rearrangements (double hit (DH) or triple hit (TH) high grade B-cell lymphomas) [4].

In DLBCL, 2[^18^F]fluoro-2-deoxy-D-glucose positron emission tomography computed tomography (^18^F-FDG PET/CT) is the current clinical standard for staging at baseline and treatment evaluation after treatment [5, 6]. Both end of treatment PET/CT (EoT-PET) scans and interim PET/CT (I-PET) are reviewed using the Lugano classification criteria (Deauville 5-point scale, DS). To improve consistency of visual assessment, quantitative cut-offs were suggested based on the maximum hepatic standardized uptake value (SUV_max_) [6]. In non-selected DLBCL patients, a negative I-PET has a high negative predictive value (NPV) for outcome, but the positive predictive value (PPV) is limited [7, 8]. One small study that included 28 *MYC* + DLBCL patients reported lower NPV values compared to non-selected DLBCL patients [9]. For EoT-PET scans, the NPV is very high in non-selected patients [10]. In *MYC* + DLBCL patients, the diagnostic performance of I-PET with regard to predicting EoT-PET response patients yielded a NPV of 79%, and a PPV of 60% [11].

Whether molecular high-risk features of DLBCL, such as *MYC* rearrangement, are correlated with PET responses has not been studied in detail. The aim of this study was to analyze the patterns of PET response in *MYC* + DLBCL at I-PET and EoT-PET patients and compare it with the response patterns of *MYC*- DLBCL patients. We also explored the changes in SUV and metabolic tumor volume (MTV) between baseline PET, I-PET, and EoT-PET scans for both *MYC* + and *MYC*- DLBCL patients.

## Material and methods

### Study population

For this analysis, we included 81 *MYC* + DLBCL patients from the multicenter phase 2 HOVON-130 trial (Eudra-CT: 2014–002,654-39) [11]. All patients included in this trial had confirmation of *MYC* rearrangement (8q24) by fluorescent in situ hybridization (FISH). *BCL2* and *BCL6* FISH results were completed when sufficient lymphoma material was available. ^18^F-FDG PET/CT scans were performed at baseline, after three cycles of R-CHOP (I-PET) and at end of treatment (EoT-PET). All patients were treated with six cycles of R-CHOP in 21-day cycles combined with lenalidomide, followed by two gifts of rituximab (R). One patient that was included in the HOVON-130 trial had no I-PET or EoT-PET scans available and was therefore excluded from this analysis. DLBCL patients (*n* = 129) from the multicenter randomized phase 3 HOVON-84 trial (EudraCT: 2006–005,174-42) who had sufficient lymphoma material available for FISH analysis and were *MYC-* served as a control group [12]. In this trial ^18^F-FDG PET/CT scans were performed at baseline, after four cycles of treatment (I-PET) and after 6–8 cycles of treatment (EoT-PET). Patients who were 65 years or younger received eight cycles of R-CHOP intensified with R (RR-CHOP) in 14-day cycles, and those above 65 years received six cycles of RR-CHOP in 14-day cycles. There was no significant difference in survival between both treatment arms, allowing for combined analysis of patients in the HOVON-84 trial.

### Qualitative image analysis

For both the HOVON-84 and HOVON-130 trials, ^18^F-FDG PET/CT scans were anonymized and uploaded to the Keosys system for web-based viewing and reporting. In total, 20 hospitals included patients for both the HOVON-84 and HOVON-130 trial, and 27 additional hospitals included patients for the HOVON-84 trial. ^18^F-FDG PET/CT scans were scanned according to local imaging guidelines. The majority of scans complied with EARL guidelines. All I-PET and EoT-PET scans were centrally reviewed according to the Deauville 5-point scale (DS) [5, 6] by two independent, experienced nuclear medicine physicians from the HOVON Imaging Working Group. DS1-3 was regarded as negative (complete metabolic response) and DS4-5 as positive (partial metabolic response or progressive metabolic disease) for both I-PET and EoT-PET. A DS of 5 was assigned when tumor SUV_max_ was ≥ 3 times higher than the hepatic SUV_max_ and/or in case of new lymphoma lesions. Reviewers used an electronic case record form with prespecified nodal (Waldeyer’s ring, cervical, supraclavicular, axillary, mediastinum, hilar, paraaortic, mesenteric, spleen, iliac, inguinal, and other) and extranodal localizations (gastrointestinal, central nervous system, skin, liver, lung, pleural, skeletal, and other). Reviewers assigned a DS for individual nodal and extranodal localizations together with a final patient-based DS. A third adjudicator resolved discrepancies between patient-based DS for both studies, and for individual nodal and extranodal localizations for the HOVON-84 study. Reviewers were blinded for clinical outcome.

For the current analysis, discrepancies between DS scores of individual nodal and extranodal localizations for the HOVON-130 study were resolved by an additional adjudication. Moreover, the presence of new lymphomatous lesions between baseline, I-PET, and EoT-PET scans was scored for both the HOVON-84 and HOVON-130 studies. The anatomical localization of new lymphoma lesions were noted according to the predefined nodal and extranodal localizations.

### Quantitative image analysis

Quantitative PET/CT analysis was performed using the ACCURATE tool [13]. Scans were included in this study if they matched the following quality criteria: both PET and low dose CT scans had to be complete, and the liver SUV_mean_ and plasma glucose should be within the ranges suggested by the European Association of Nuclear Medicine guidelines [14]. If the liver SUV_mean_ was outside the suggested ranges, but the total image activity was between 50 and 80% of the total injected activity; these scans were still included.

To assess the changes in uptake and MTV between baseline, I-PET, and EoT-PET for I-PET and EoT-PET-positive lesions, we extracted the MTV and SUV_max_ of these lesions. Baseline MTV of these lesions was calculated using the fixed SUV4.0 segmentation method [15, 16]. For PET-positive lesions at I-PET and EoT-PET, MTV was calculated using the method with the most complete tumor segmentation without oversegmentation of non-tumor regions, which could be a fixed threshold (SUV4.0 or SUV2.5), a relative threshold (41%max or 50%peak) or an averaging method (MV2 or MV3). For the MV2 method, voxels detected by ≥ 2 methods (out of SUV4.0, SUV2.5, 41%max, and 50%peak) were selected, and for MV3 method, voxels detected by ≥ 3 methods were selected. For the generated MTV at baseline, I-PET and EoT-PET non-tumor ^18^F-FDG avid regions (e.g., bladder, kidney) adjacent to lymphoma lesions were manually removed. All delineations were performed under supervision of an experienced nuclear medicine physician. An increase of 30% or 1.3 units in SUV_max_, whichever is largest, as cut-off for the smallest detectable change in SUV_max_. For MTV, we also applied a cut-off of 30% increase [17].

### Statistical analysis

The primary endpoint was 2-year overall survival (OS), defined as time from registration to death. Patients still alive were censored at date of last contact.

The Chi-square test for independence was used to compare individual IPI components and patient characteristics, cell of origin, and the differences between PET positive and PET negative proportions at I-PET and EoT-PET between *MYC* + DLBCL and *MYC*- DLBCL subgroups. As a sensitivity analysis, *MYC* + patients were matched with *MYC*- DLBCL patients based on IPI, Ann Arbor stage, and extranodal involvement. The Chi-square test for independence was used to compare the differences between PET positive and PET negative proportions at I-PET and EoT-PET in this subgroup. Moreover, the Chi-square test for independence was used to compare proportions of patients with new lesions, increased SUV, and increased MTV between *MYC* + DLBCL and *MYC*- DLBCL subgroups for EoT-PET compared to I-PET and baseline and I-PET compared to baseline, respectively. A *p* value of less than 0.05 was considered statistically significant.

Survival curves were obtained with Kaplan–Meier (KM) analyses stratified for PET response at I-PET and EoT-PET for both *MYC* + and *MYC-* DLBCL patients. KM curves were compared with log-rank tests. The predictive value of I-PET and EoT-PET was assessed by calculating diagnostic measures (sensitivity, specificity, positive, and negative predictive values (PPV and NPV), respectively) using 2 × 2 contingency tables.

The predictive value of other cut-off values of the DS (DS1-2 vs 3–5 and DS1-4 vs 5) for I-PET and EoT-PET were evaluated in sensitivity analyses by calculation of the sensitivity, specificity, PPV, and NPV.

## Results

Baseline patient characteristics of *MYC* + DLBCL and *MYC*- DLBCL are presented in Table 1. In *MYC* + DLBCL patients, advanced stage disease (*p* = 0.010), higher lactate dehydrogenase (LDH) levels (*p* = 0.097), extranodal involvement (*p* = 0.003), and GCB subtype (*p* = 0.0004) were more frequent compared to *MYC*- DLBCL patients.

**Table 1:** Baseline patient characteristics of *MYC* + and *MYC*- DLBCL patients

	MYC + (*n* = 81)	MYC- (*n* = 129)
Age		
Median (IQR)	63 (54–72)	66 (58–73)
≤ 60 years	37 (45.7%)	39 (30.2%)
> 60 years	44 (54.3%)	90 (69.8%)
Sex		
Male	55 (67.9%)	62 (48.1%)
Female	26 (32.1%)	67 (51.9%)
Ann Arbor Stage		
2	7 (8.6%)	20 (15.5%)
3	11 (13.6%)	35 (27.1%)
4	63 (77.8%)	74 (57.4%)
LDH		
Normal	20 (24.7%)	50 (38.8%)
> normal	56 (69.1%)	79 (61.2%)
Unknown	5 (6.2%)	
Extranodal localizations		
≤ 1	32 (39.5%)	79 (61.2%)
> 1	49 (60.5%)	50 (38.8%)
WHO performance status		
0	48 (59.3%)	79 (61.2%)
1	26 (32.1%)	36 (27.9%)
2	5 (6.2%)	14 (10.9%)
3	2 (2.5%)	
IPI		
Low	9 (11.1%)	27 (20.9%)
Low-intermediate	18 (22.2%)	29 (22.5%)
High-intermediate	35 (43.2%)	40 (31.0%)
High	19 (23.5%)	33 (25.6%)
COO		
GCB	62 (76.5%)	67 (51.9%)
Non-GCB	8 (9.9%)	42 (32.6%)
Not evaluable	11 (13.6%)	20 (15.5%)
MYC status		
Negative		129 (100%)
Single hit	22 (27.2%)	
Double hit/triple hit	52 (64.2%)	
MYC + (BCL2 and BCL6 unknown)	7 (8.6%)	

Abbreviations: *IQR* interquartile range, *LDH* lactate dehydrogenase, *WHO* World Health Organisation, *IPI* International Prognostic Index, *COO* cell of origin

### PET response rates

**Fig. 1:**
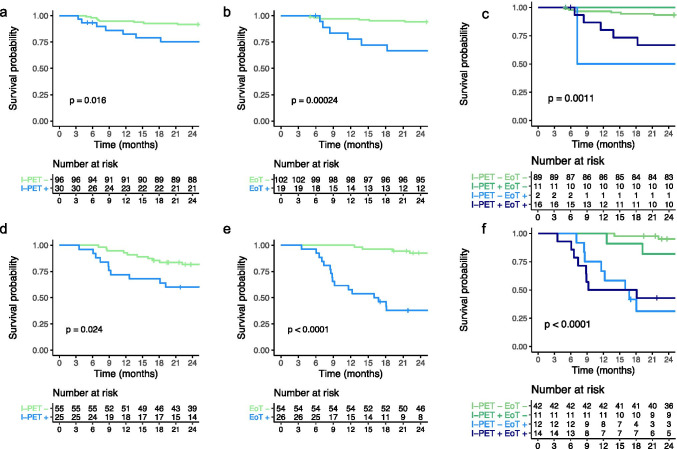
Kaplan–Meier survival curves for time to progression stratified by interim PET and end of treatment PET response for *MYC* + and MYC*-* DLBCL patients. **a**–**c** Survival curves for *MYC*- DLBCL patients stratified by (**a**) interim PET response, (**b**) end of treatment response, (**c**) a combination of interim- and end of treatment response. **d**–**f** Survival curves for *MYC* + DLBCL patients stratified by (**d**), interim PET response, (**e**) end of treatment response, and (**f**) a combination of interim- and end of treatment response

For the *MYC* + DLBCL patients, 80 I-PET scans and 80 EoT-PET scans were centrally reviewed. At I-PET, 23 out of 80 patients were PET positive (28.8%, Table 2). Two-year OS for I-PET negative patients was 81.6%, and 60% for I-PET positive patients (*p* = 0.024; Fig. 1d). At EoT-PET, 26 out of 80 patients were PET positive (32.5%). Thirteen out of 23 I-PET-positive patients remained PET-positive at EoT and 13 out of 56 (23%) I-PET-negative patients converted to PET positivity at EoT. Two-year OS for EoT-PET-negative patients was 92.5%, and 37.8% for EoT-PET-positive patients (*p* < 0.001, Fig. 1e).

**Table 2:** Response rates of *MYC* + and *MYC*- DLBCL patients on interim PET and end of treatment PET

		EoT-	EoT +	No EoT
*MYC* +	I-PET-	43	13	1
	I-PET +	10	13	
	No I-PET	1		
*MYC*-	I-PET-	89	2	5
	I-PET +	11	16	3
	No I-PET	2	1	
*MYC*-matched	I-PET-	67	1	
	I-PET +	8	16	

Abbreviations: *DLBCL* diffuse large B-cell lymphoma, *I-PET* interim PET, *EoT* end of treatment PET, *I-PET-/EoT-* Deauville score 1-3, *I-PET* + */EoT* + Deauville score 4-5

For the *MYC*- DLBCL patients, 126 I-PET scans and 121 EoT-PET scans were centrally reviewed. At I-PET, 30 out of 126 patients were PET positive (23.8%), which was comparable to the I-PET positivity rate of *MYC* + DLBCL patients (*p* = 0.399). Two-year OS for I-PET-negative patients was 91.7%, and 75.4% for I-PET-positive patients (*p* = 0.016, Fig. 1a). At EoT, 19 out of 121 patients were PET positive (15.7%), which was significantly lower than the EoT PET positivity rate in *MYC* + DLBCL patients (*p* = 0.004). Sixteen out of 30 I-PET positive patients remained PET-positive at EoT-PET and 2 out of 91 (2%) I-PET-negative patients with an EoT-PET scan converted to PET-positivity at EoT. Two-year OS for EoT-negative patients was 94.1%, and 66.7% for EoT-positive patients (*p* < 0.001, Fig. 1b).

*MYC* status and PET response at I-PET and EoT-PET were significantly associated (*χ*^2^ [3, *n* = 197] = 17.4, *p* < 0.001). For *MYC* + DLBCL patients, fewer patients were PET-negative at I-PET and EoT-PET (*p* < 0.001), and more patients were I-PET-negative and EoT-PET-positive (*p* = 0.002) compared to expected frequencies. Vice versa, for *MYC*- DLBCL patients, more patients were PET negative at I-PET and EoT, and fewer patients were I-PET-negative and EoT-PET-positive. No differences in frequencies of I-PET-positive and EoT-PET-negative and I-PET-positive and EoT-PET-positive groups were observed between *MYC* + and *MYC-* DLBCL patients.

Upon matching 79 *MYC* + with 92 *MYC*- DLBCL patients for IPI, Ann Arbor stage, and extranodal involvement, *MYC* status and PET response were still significantly associated (*χ*^2^[3, *n* = 171] = 14.9, *p* = 0.002). Twenty-four out of 92 (26.1%) of the *MYC*- DLBCL patients in this matched subset were I-PET-positive, whereas 17 out of 92 (18.5%) of the *MYC*- DLBCL patients were EoT-PET-positive (Table 2). I-PET response rates of *MYC*- DLBCL patients were comparable (*p* = 0.719), but EoT-PET response rates were significantly lower (*p* = 0.008) compared to *MYC* + DLBCL patients.

Using the DS1-2 vs 3–5 cut-off resulted in more I-PET- and EoT-PET-positive patients in both *MYC* subgroups (Supplemental Table 1), resulting in lower specificity and PPV and higher sensitivity (Supplemental Table 2). Using the DS1-4 vs 5 cut-off resulted in fewer I-PET- and EoT-PET-positive patients (Supplemental Table 3), leading to higher PPV and specificity and lower sensitivity (Supplemental Table 4).

### New localizations

**Fig. 2:**
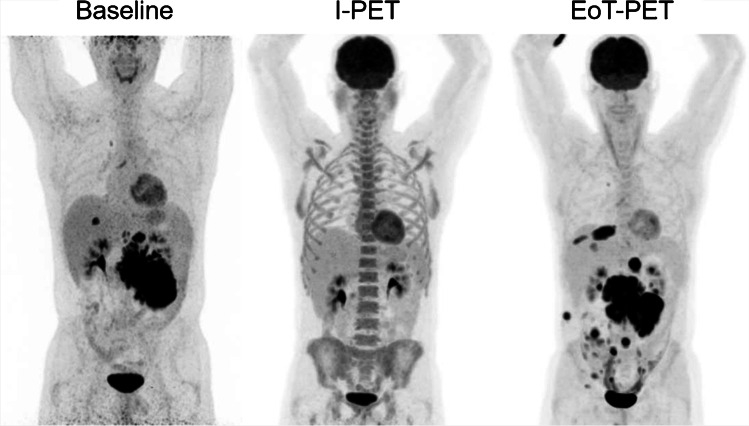
Maximum intensity projections of a *MYC* + DLBCL patient that presented with new PET positive lesions at EoT-PET that were not initially involved. Images are scaled using a SUV0-10 scale

Twenty-one out of 26 EoT-PET-positive *MYC* + DLBCL patients had new PET-positive localizations at EoT-PET compared to I-PET. Nine EoT-PET-positive patients had new PET-positive localizations that were not initially involved at baseline (1 SH, 6 DH, and 2 TH DLBCL patients) (Fig. 2). Eight out of nine patients had multiple new PET-positive lesions; one patient had a single new PET-positive lesion at EoT-PET compared to baseline (Table 3). Six of these patients presented with new lesions both at nodal and extranodal sites at EoT-PET, two had new extranodal lesions, and one had a single new nodal PET-positive localization. All nine patients had both nodal and extranodal localizations at baseline. In comparison, three out of 19 *MYC*- DLBCL patients had a single new PET-positive extranodal localization at EoT compared to I-PET (*MYC* + : 21 out of 26; *p* < 0.001). One of these patients had one new extranodal localization at EoT that was not initially involved at baseline (*MYC* + : 9 out of 26; *p* = 0.036).

**Table 3:** New PET positive localizations that were not initially involved at baseline

Patient	MYC status	New localisations at EoT-PET
1	Positive	Cervical, thyroid
2	Positive	Mesenteric, paraaortic, iliac, pleural, subcutaneous
3	Positive	Fascia Gerota, perirenal
4	Positive	Mediastinum, peritoneum
5	Positive	Lung, retroperitoneum, left kidney
6	Positive	Skeletal, spleen
7	Positive	Paraaortic, iliac, kidney, mons pubis
8	Positive	Cervical
9	Positive	Iliac, uterus,
10	Negative	Central nervous system

### Diagnostic performance I-PET and EoT-PET in MYC + and MYC- patients

Using 2-year OS as outcome, the NPV of I-PET and EoT-PET was high (range 81.8–94.1%), and higher for both I-PET and EoT-PET in the *MYC*- DLBCL patients compared to the *MYC* + DLBCL patients (Table 4). NPV was highest at EoT-PET for *MYC*- DLBCL patients (94.1%). PPV was generally low (range 31.6–61.5%), but higher for the *MYC* + DLBCL patients compared to *MYC-* DLBCL patients for both I-PET and EoT-PET. PPV was highest at EoT-PET for *MYC* + patients (61.5%).

**Table 4:** Diagnostic performance of interim PET and end of treatment PET stratified for *MYC* + and *MYC*- DLBCL patients using 2-year overall survival as outcome parameter

	I-PET		EoT-PET	
	*MYC* + (*n* = 80)	*MYC*- (*n* = 126)	*MYC* + (*n* = 80)	*MYC*- (*n* = 121)
Sensitivity	50.0 (27.2–72.8)	46.7 (21.3–73.4)	80.0 (56.3–94.3)	50.0 (21.1–78.9)
Specificity	75.0 (62.1–85.3)	79.3 (70.6–86.4)	83.3 (71.5–91.7)	88.1 (80.5–93.5)
Positive predictive value	40.0 (26.4–55.3)	23.3 (13.7–36.9)	61.5 (46.6–74.6)	31.6 (17.7–49.7)
Negative predictive value	81.8 (73.9–87.7)	91.7 (87.2–94.7)	92.6 (83.8–96.8)	94.1 (90.0–96.6)

Abbreviations: *DLBCL* diffuse large B-cell lymphoma, *I-PET* Interim PET, *EoT* End of treatment PET

### Change in uptake and metabolic tumor volume

For the *MYC* + DLBCL subgroup, 13 patients were PET-positive at both I-PET and EoT-PET, whereas for the *MYC-* DLBCL patients, 16 were PET-positive at both I-PET and EoT-PET. At patient level, 62.5% of the I-PET- and EoT-PET-positive *MYC* + DLBCL patients had higher MTV at EoT than at I-PET, whereas relatively fewer (38.5%, *p* = 0.140) of the *MYC*- DLBCL patients with PET-positive results showed higher MTV at EoT compared to I-PET.

For the *MYC* + DLBCL patients, four patients out of 23 patients (17.4%) showed increased lesional uptake, and two patients (8.7%) showed increased lesional MTV at I-PET compared to baseline (Table 5). At EoT-PET, 21 out of 26 patients (80.8%) showed increased lesional MTV and uptake compared to I-PET, whereas 11 out of 26 patients (42.3%) showed increased lesional uptake, and nine (34.6%) patients showed increased lesional MTV at EoT-PET compared to baseline (Table 5).

**Table 5:** Number of I-PET or EoT-PET positive patients that showed lesional increased SUV or MTV during treatment compared to baseline or I-PET stratified for *MYC* + and *MYC-* DLBCL patients

		*MYC* +		*MYC*-	
		Increase	No increase	Increase	No increase
MTV	Baseline vs I-PET	2	21	0	30
	I-PET vs EoT-PET	21	5	8	11
	Baseline vs EoT-PET	9	17	1	18
SUV	Baseline vs I-PET	4	19	1	29
	I-PET vs EoT-PET	21	5	6	13
	Baseline vs EoT-PET	11	15	2	17

Data are presented as number of patients

Abbreviations: *MTV* metabolic tumor volume, *SUV* standardized uptake value, *vs* versus

For the *MYC*- DLBCL patients, one patient out of 30 patients (3.3%) showed increased uptake at lesional level (*MYC* + : 4 out of 23; *p* = 0.085), and no patients showed increased MTV at I-PET compared to baseline (*MYC* + : 2 out of 23;* p* = 0.103). At EoT-PET, six out of 19 patients (31.6%) showed increased lesional uptake (*MYC* + : 21 out of 26; *p* = 0.001) and eight patients (42.1%) showed increased lesional MTV compared to I-PET (*MYC* + : 21 out of 26; *p* = 0.008), whereas two out of 19 patients (10.5%) showed increased lesional uptake (*MYC* + : 11 out of 26; *p* = 0.022), and one patient (5.3%) showed increased lesional MTV compared to baseline (*MYC* + : 9 out of 26; *p* = 0.021).

## Discussion

This study shows that *MYC* + DLBCL patients have a different PET response compared to *MYC*- DLBCL patients. They experience progressive disease during treatment after I-PET-negative assessment more frequently. Moreover, a substantial part of the *MYC* + DLBCL patients present with new PET positive lesions at previously uninvolved sites.

In general, I-PET could be a suitable technique to ensure effectiveness of treatment and to exclude the possibility of progression for DLBCL patients [6]. *MYC* + DLBCL patients have a poor prognosis and relapses are frequent, stressing the need for identification of early markers that reliably predict poor outcomes in this patient population. However, this study showed that the role of I-PET is less evident in this *MYC* + patient subgroup, as a negative I-PET assessment results in a positive EoT scan in 22.8% of the patients. We previously reported that in *MYC* + patients, I-PET response has limited predictive value for EoT-PET response [11]. Nevertheless, in this study, we showed that I-PET can significantly differentiate between responders and non-responders in *MYC* + DLBCL patients when using 2-year OS as outcome parameter. This is in contrast with the results of 2 other studies, which showed that I-PET did not predict survival in *MYC* + DLBCL patients [9, 18]. Moreover, we showed that I-PET can also significantly differentiate responders and non-responders in *MYC-* patients. There are no studies that looked at the predictive value of I-PET in *MYC-* DLBCL patients only. But these results are in line with previous studies, where *MYC* status was not assessed [7, 8, 19, 20].

In our study, 28.8% of the *MYC* + patients had positive I-PET scans. This PET-positivity rate was comparable with the PET-positivity rate of *MYC*- patients (23.8%), and with PET-positivity rates reported in a recent individual patient data analysis [8]. However, Dunleavy and colleagues reported that 70.8% of *MYC* + DLBCL patients treated with dose-adjusted etoposide, prednisone, vincristine, cyclophosphamide, doxorubicin, and rituximab (EPOCH-R) were PET positive after two cycles of treatment [18]. This positivity rate is much higher than our PET-positivity rates, and the PET-positivity rates of a smaller study incorporating I-PET in *MYC* + DLBCL patients [9]. This higher PET-positivity rate could not be explained by the DH/TH vs SH ratio, by lower survival rates or by different PET positivity criteria, since all studies used the DS taxonomy. Results of another study that included 51 primary B-cell lymphoma patients treated with dose-adjusted EPOCH-R suggested treatment-related inflammation with this treatment-regimen [21]. As the interval between the previous cycle of chemotherapy and I-PET is usually short, this could explain the higher PET-positivity rates in the study of Dunleavy et al. [18]. However, the I-PET-positivity rates in a recent trial, where half of the DLBCL patients were treated with dose-adjusted EPOCH-R [20], did not show higher I-PET positivity rates compared to other studies, contradicting this hypothesis.

Richter et al. showed that *MYC* + DLBCL patients, with or without *BCL2* and/or *BCL6* rearrangements, were overrepresented (6/25 vs 21/241) in the I-PET positive group, using the delta SUV response criterion, compared to the I-PET negative group [22]. Furthermore, Yuan et al. also showed that *MYC* rearrangements were significantly positively correlated with a positive I-PET using delta SUV response criterion, both after 1 and 2 cycles of treatment [23]. These findings were not confirmed in our study: when applying the delta SUV response criterion, 11 *MYC* + DLBCL patients were positive at I-PET (13.8%), which was not significantly higher than the proportion of I-PET positive patients in *MYC*- DLBCL patients (9.6%, *n* = 12).

The EoT-PET positivity rates were significantly higher in the *MYC* + DLBCL patient group compared to the *MYC*- DLBCL patient group. As shown by our sensitivity analysis, the significant association between *MYC* status and PET response remained significant when matching patients on baseline risk, indicating that this higher EoT-PET positivity rate is associated with their *MYC* status, and not the higher baseline risk of these patients. To the best of our knowledge, no other study reported the EoT-PET positivity rate for solely *MYC* + DLBCL patients, nor did any study investigate the increase in SUV or MTV between baseline, I-PET, and EoT-PET. Our findings therefore need to be validated in other studies in which both an I-PET and EoT-PET for *MYC* + DLBCL patients will be performed.

Our study shows that the NPV was high at I-PET and EoT-PET for *MYC-* DLBCL patients. This NPV is higher than the NPV of I-PET of most DLBCL studies that included both *MYC-* and *MYC* + DLBCL patients [7], indicating that further stratification of patients using *MYC* status improves the NPV of I-PET and EoT-PET scans for DLBCL patients. The PPV of both I-PET and EoT-PET is lower, especially for *MYC*- patients. For *MYC* + patients, the PPV at EoT-PET is relatively high.

To the best of our knowledge, this is the first study to make a head-to-head comparison of PET response patterns of *MYC* + DLBCL and *MYC*- DLBCL patients. One of the strengths of this study is the fact that the PET scans and pathology data were centrally reviewed. The uniform interpretation of scans and pathology in both studies resulted in high-quality data. Moreover, with a total of 214 patients included, of which 82 had a *MYC* translocation, the sample size of this study is fairly large allowing firm conclusions. We choose OS as outcome parameter, instead of the more commonly used progression free survival (PFS) because progression for the *MYC* + DLBCL patients was often confirmed using the EoT-PET results. Therefore, almost all *MYC* + DLBCL patients with a positive EoT-PET had a PFS event at EoT-PET. Furthermore, SH and DH/TH DLBCL patients were included in our *MYC* + DLBCL group. Unfortunately, our subgroups of SH and DH/TH patients were too small to draw conclusions per subgroup. This study has some limitations. First, *MYC* + patients received intensified chemotherapy compared to the *MYC*- patients. This treatment of *MYC* + patients is believed to be more effective than R-CHOP without intensification. Therefore, if these patients would receive R-CHOP without intensification, the patterns of PET response between *MYC* + and *MYC*- groups could be different. Secondly, the timing of I-PET differed between subgroups. A recent individual patient data analysis showed that I-PET positivity using DS criteria at I-PET3 and I-PET4 was almost identical (21.7 vs 21.2%) [8], implying that the I-PET-positivity rate for *MYC*- DLBCL patients would be similar after three cycles of treatment. However, a small effect of timing cannot be precluded in our results. Thirdly, new PET + localizations at EoT were not biopsy proven, but at least one new localization in each patient was located at sites that are not suspicious for infections or other causes. Lastly, not all patients in the HOVON-84 study had enough biopsy material to confirm their *MYC* status, which could have led to selection bias. However, when matching *MYC* + and *MYC*- patients on baseline characteristics, we still found similar results, implying that selection bias did not influence our results to a large extent.

Future studies should focus on the potential of other quantitative PET measures or molecular biomarkers to stratify “good” and “poor” responders within *MYC* + DLBCL patients. Robust and easy to use biomarkers for early identification of poor responders in this patient group are essential and an integrative approach with both molecular data and quantitative PET metrics could improve prediction of prognosis and guide choice of therapies.

## Conclusion

*MYC* + DLBCL patients demonstrate different response patterns compared to *MYC*- DLBCL patients with more progressive disease during treatment after I-PET negative assessment, with more nodal and extranodal lesions at sites that were not initially involved.

## Supplementary Information

Below is the link to the electronic supplementary material.

ESM (DOCX 19.1 KB)

## Data Availability

The datasets generated during and/or analyzed during the current study are available from the corresponding author on reasonable request.
