# Supplementary material for: Aberrant patterns of PET response during treatment for DLBCL patients with MYC gene rearrangements
Source: Eur J Nucl Med Mol Imaging. 2021 Sep 2;49(3):943–52. doi: 10.1007/s00259-021-05498-7 (PMC8803795; doi:10.1007/s00259-021-05498-7)
Supplement: Supplementary file 1 — ESM (DOCX 19.1 KB) [file 259_2021_5498_MOESM1_ESM.docx]

**Supplementary information**

**Article title:** Aberrant patterns of PET response during treatment for DLBCL patients with MYC gene rearrangements

**Journal name:** European Journal of Nuclear Medicine and Molecular Imaging

**Author names**: J.J. Eertink, A.I.J. Arens, J.E. Huijbregts, F. Celik, B. de Keizer, S. Stroobants, D. de Jong, S.E. Wiegers, G.J.C. Zwezerijnen, C.N. Burggraaff, R. Boellaard, H.C.W. de Vet, O.S. Hoekstra, P.J. Lugtenburg, M.E.D. Chamuleau and J.M. Zijlstra **Affiliation and e-mail address of the corresponding author:** Amsterdam UMC, Vrije Universiteit Amsterdam, department of Hematology, Cancer Center Amsterdam, De Boelelaan 1117, Amsterdam, Netherlands, e-mail: [j.zijlstra@amsterdamumc.nl](mailto:j.zijlstra@amsterdamumc.nl)

Supplementary Table 1. Response rates of n=81 *MYC*+ and n=129 *MYC*- DLBCL patients on interim PET and End of Treatment PET using DS1-2 vs 3-5 response criteria.

|  |  | EoT- | EoT+ | No EoT |
| --- | --- | --- | --- | --- |
| *MYC*+ | **I-PET-** | 30 | 10 | 1 |
|  | **I-PET+** | 12 | 27 |  |
|  | **No I-PET** | 1 |  |  |
| *MYC*- | **I-PET-** | 73 | 1 | 4 |
|  | **I-PET+** | 14 | 22 | 4 |
|  | **No I-PET** | 2 | 1 |  |

*Abbreviations: DLBCL: diffuse large B-cell lymphoma, I-PET: interim PET, EoT: End of Treatment PET*

|  | I-PET | | EoT-PET | |
| --- | --- | --- | --- | --- |
|  | *MYC*+ (n=80) | *MYC*- (n=126) | *MYC*+ (n=80) | *MYC*- (n=121) |
| Sensitivity | 65.0 (40.8-84.6) | 53.3 (26.6-78.7) | 95.0 (75.1-99.9) | 50.0 (21.1-78.9) |
| Specificity | 56.7 (43.2-69.4) | 65.8 (56.2-74.5) | 70.0 (56.8-81.1) | 78.9 (70.0-86.1) |
| Positive predictive value | 33.3 (24.5-43.5) | 17.4 (10.9-26.5) | 51.4 (41.5-61.2) | 20.7 (11.8-33.8) |
| Negative predictive value | 82.9 (72.0-90.2) | 91.2 (85.7-94.8) | 97.7 (86.1-99.7) | 93.5 (89.0-96.2) |

Supplementary Table 2. Diagnostic performance of interim PET and End of Treatment PET stratified for *MYC+* and *MYC*- DLBCL patients using DS1-2 vs 3-5 response criteria.

*Abbreviations: DLBCL: diffuse large B-cell lymphoma, I-PET: interim PET and EoT: End of Treatment PET*

Supplementary Table 3. Response rates of n=81 *MYC*+ and n=129 *MYC*- DLBCL patients on interim PET and End of Treatment PET using DS1-4 vs 5 response criteria.

|  |  | EoT- | EoT+ | No EoT |
| --- | --- | --- | --- | --- |
| *MYC*+ | **I-PET-** | 59 | 13 | 1 |
|  | **I-PET+** | 1 | 6 |  |
|  | **No I-PET** | 1 |  |  |
| *MYC*- | **I-PET-** | 109 | 3 | 7 |
|  | **I-PET+** | 2 | 4 | 1 |
|  | **No I-PET** | 2 | 1 |  |

*Abbreviations: DLBCL: diffuse large B-cell lymphoma, I-PET: interim PET, EoT: End of Treatment PET*

Supplementary Table 4. Diagnostic performance of interim PET and End of Treatment PET stratified for *MYC+* and *MYC*- DLBCL patients using DS1-4 vs 5 response criteria.

|  | I-PET | | EoT-PET | |
| --- | --- | --- | --- | --- |
|  | *MYC*+ (n=80) | *MYC*- (n=126) | *MYC*+ (n=80) | *MYC*- (n=121) |
| Sensitivity | 20.0 (5.8-43.7) | 33.3 (11.8-61.6) | 70.0 (45.7-88.1) | 41.7 (15.2-72.3) |
| Specificity | 95.0 (86.1-99.0) | 98.2 (93.6-99.8) | 91.7 (81.6-97.2) | 98.2 (93.5-99.8) |
| Positive predictive value | 57.1 (24.6-84.5) | 71.4 (34.7-92.2) | 73.7 (53.6-87.2) | 71.4 (35.2-92.0) |
| Negative predictive value | 78.1 (74.0-81.7) | 91.6 (88.4-94.0) | 90.2 (82.4-94.7) | 93.9 (90.5-96.1) |

*Abbreviations: DLBCL: diffuse large B-cell lymphoma, I-PET: interim PET and EoT: End of Treatment PET*
